# Field emission characteristics of zinc oxide nanowires synthesized by vapor-solid process

**DOI:** 10.1186/1556-276X-9-70

**Published:** 2014-02-11

**Authors:** Shou-Yi Kuo, Hsin-I Lin

**Affiliations:** 1 Department of Electronic Engineering, Chang Gung University, Taoyuan, Taiwan; 2 Advanced Optoelectronic Technology Center, National Cheng-Kung University, Tainan 701, Taiwan

**Keywords:** ZnO nanowires, 1D nanostructures, Field emission, Near band edge

## Abstract

Vertically aligned ZnO nanowire (NW) arrays have been synthesized on silicon substrates by chemical vapor deposition. The growth of ZnO NWs may be dominated by vapor-solid nucleation mechanism. Morphological, structural, optical, and field emission characteristics can be modified by varying the growth time. For growth time that reaches 120 min, the length and diameter of ZnO NWs are 1.5 μm and 350 nm, respectively, and they also show preferential growth orientation along the *c*-axis. Room-temperature photoluminescence spectra exhibit a sharp UV emission and broad green emission, and the enhanced UV-to-green emission ratio with increasing growth time might originate from the reduced concentration of surface defects. Furthermore, strong alignment and uniform distribution of ZnO NWs can also effectively enhance the antireflection to reach the average reflectance of 5.7% in the visible region. The field emission measurement indicated that the growth time plays an important role in density- and morphology-controlled ZnO NWs, and thus, ZnO NWs are expected to be used in versatile optoelectronic devices.

## Background

Recently, semiconductor one-dimensional (1D) nanostructures have been attracting much attention in fundamental research and in potential applications for nanodevices. There are numerous studies on 1D nanostructures of Si, Ge, and III-V and also on oxide systems such as tin oxide (SnO_2_), silicon oxide (SiO_2_), indium tin oxide (ITO), zinc oxide (ZnO), and aluminum oxide (Al_2_O_3_). Among them, ZnO has been expected to be one of the most important optoelectronic materials with piezoelectricity, biocompatibility, wide bandgap (approximately 3.37 eV), and large exciton binding energy (approximately 60 meV) at room temperature [1,2]. Due to their exceptional physical and chemical properties, 1D ZnO nanostructures, such as nanorods, nanowires (NWs), nanotubes, and nanoneedles, are very attractive as well. Arrays of vertically aligned ZnO nanostructures are considered to be a promising candidate for applications in blue UV light emitters, field emission devices, high-efficiency photonic devices, photovoltaic devices, and biosensors [3-10]. So far, various kinds of high-quality and well-aligned 1D ZnO nanostructures have been realized using vapor-phase transport, metal-organic vapor-phase epitaxy, pulsed laser deposition, and wet chemistry methods [11-15]. Vapor–liquid-solid (VLS) and vapor-solid (VS) processes have been employed by many researchers for the growth of 1D ZnO nanostructures because of its simple procedure and relatively low cost. The VLS model has been used to explain the growth mechanism at high temperatures, while the VS model dominates at low temperatures. From the viewpoint of applications, a high-temperature process might damage or deteriorate optoelectronic devices. A low-temperature VS process would be more suitable for the integration of 1D ZnO-based devices. Besides, the important characteristic of the field emission of ZnO NWs is rarely investigated, which could be a candidate for field electron emitters due to their high aspect ratios, negative electron affinity, and mechanical and chemical stability. In this paper, we report a simple synthesis of ZnO NWs on a silicon substrate using the VS process at a relatively low growth temperature (550°C).

## Methods

ZnO NWs were synthesized in a horizontal tube furnace system equipped with a 90-cm-long quartz tube, three-zone heating system, gas inlet, and pump out. A 1 × 1 cm-sized, n-type Si(100) has been used as the deposited substrate. Before being loaded, the silicon substrate was etched using hydrofluoric acid and cleaned ultrasonically with ethanol and deionized water. After finishing substrate pretreatment, the silicon substrates were coated with 8-nm-thick Au films as buffer layer by a DC sputter. An alumina boat loaded with zinc powder (100 mesh, 99.99%) was placed at the center of the quartz tube, and the silicon substrates were placed a few centimeters downstream from the source. After loading, the quartz tube was heated up to 550°C under a constant high-purity Ar gas (150 sccm, 99.99%). The temperature was held at the peak temperature for 60, 90, 120 min, respectively. After evaporation, the system was naturally cooled down to room temperature under flowing argon gas.

The structure of as-grown samples was analyzed by X-ray diffraction (XRD; D5005, Siemens AG, Munich, Germany) using CuKα1 radiation. The morphology and microstructure were investigated by scanning electron microscopy (SEM; S-4300, Hitachi, Tokyo, Japan). Photoluminescence (PL) measurement was performed at room temperature using *λ* = 325 nm of excitation of a He-Cd laser source (IK3401R-F, Kimmon Koha Co., Ltd., Tokyo, Japan). Field emission was measured at room temperature in a vacuum ambient of 3.5 × 10^5^ Torr. The distance between the anode and the tip of the ZnO NWs was 18 μm, and the emission current was monitored with a Keithley 237 electrometer (Cleveland, OH, USA) and recorded at 1.0-s intervals by applying a sweep step of 10 V.

## Results and discussion

**Figure 1:**
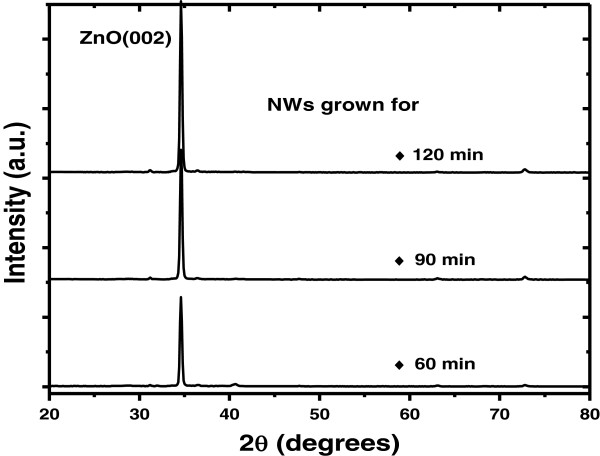
XRD patterns of ZnO NWs grown at 550°C for 60, 90, and 120 min, respectively.

XRD was used to acquire the crystallographic property of the ZnO NWs. Shown in Figure 1 are the XRD patterns of NWs grown at 550°C for 60, 90, and 120 min, respectively. Obviously, only the diffraction peak of ZnO(002) appears in the XRD profiles without the existence of secondary phases and clusters. This indicates that the ZnO NWs are preferentially oriented in the *c*-axis direction. While increasing the growth duration from 60 to 120 min, the intensity of ZnO(002) diffraction plane increased as well. As expected, the highly enhanced (002) peak can be regarded as a result of enhanced NW growth in both vertical and lateral orientations.

**Figure 2:**
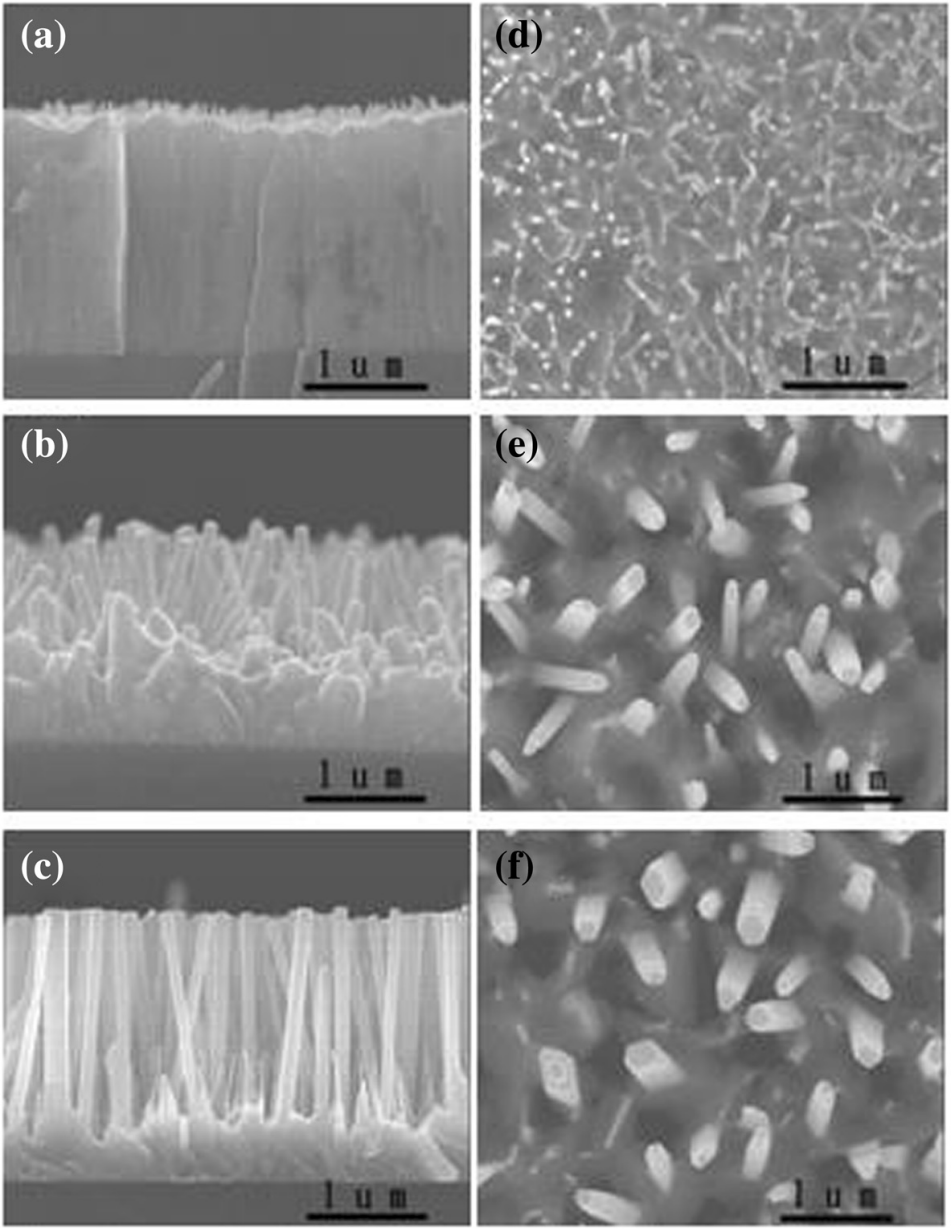
**Cross-sectional and top-view FESEM images of ZnO NWs grown at different growth times. (a, d)** NWs grown for 60 min, **(b, c, e, f)** from a Zn source at 550C for (a) 60 min (b) 90 min, and (c) 120 min of reaction times.

Figure 2a,b,c,d,e,f shows the cross-sectional and plane-view FESEM images of the ZnO NWs for different growth durations. It is notable that both the average length and diameter of the NWs increase as the growth time is increased. In addition, the areal densities of ZnO NWs are 5.2 × 10^9^, 2.9 × 10^9^, and 1.8 × 10^9^/cm^2^ with growth time of 60, 90, and 120 min, respectively. By varying the growth time from 60 to 120 min, the diameters of ZnO NWs increased from several tens to several hundreds of nanometers, and the lengths increased from 200 nm to 1.5 μm accordingly. It is also noteworthy that the ZnO NWs were almost aligned to the substrate surface. These observations are consistent with the XRD results. In a typical metal-catalyzed VLS mechanism, nanosized metal clusters play a critical role in forming liquid droplets that adsorb the gas-phase reactants where nanorod growth occurs. Hence, metallic nanoparticles with spherical shape are commonly found at the end of nanorods grown by the metal-catalyzed VLS method. Since no metallic particle was observed on the top of the ZnO NWs, we could rule out the possibility of a VLS-like mechanism and claim that the VS model dominates the nanowire growth.

**Figure 3:**
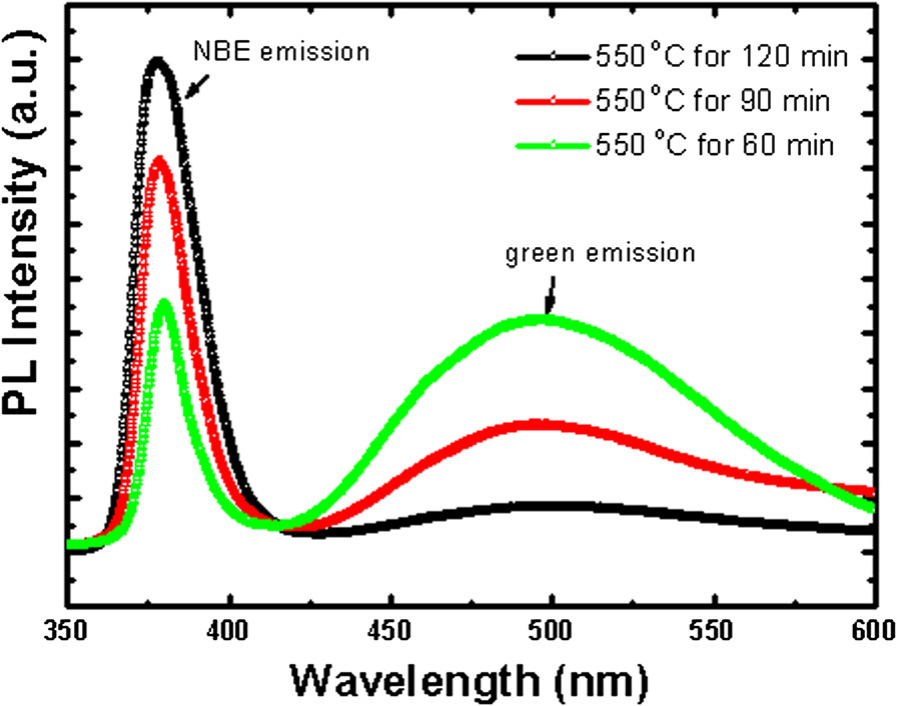
P**hotoluminescence spectra of ZnO NWs grown at different growth times.** Near-band-edge emission and green emission are labeled.

Photoluminescence of the obtained ZnO NWs synthesized at different growth times was also investigated at room temperature, and the results are shown in Figure 3. The PL spectra consist of a sharp and strong UV emission peak centered at about 380 nm and a weak green emission centered at about 500 nm. The UV emission is attributed to the near-band-edge (NBE) emission, and the green emission is related to the intrinsic defects in the ZnO samples. When the growth time increased, the intensity of NBE emission (*I*_NBE_) also increased while the green emission (*I*_green_) decreased. Since ZnO NWs were fabricated under a fixed growth temperature, the improvement of crystal quality might play a minor role. Thus, an increase in the NBE-to-green emission ratio with increasing growth time could result from the reduced concentration of surface defects. Generally, the green emission is attributed to single ionized oxygen vacancies (*V*_o_) [16]. Recently, it has been recognized that the surface states that originated from large surface-to-volume ratios seriously influence the PL features in nanomaterials. As manifested in the SEM images, the average diameter becomes smaller with decreasing growth time. Having a larger surface-to-volume ratio in nanostructures means a larger density of surface states. Therefore, higher surface states of ZnO NWs with a smaller diameter can be responsible for the origin of the enhanced green emission.

**Figure 4:**
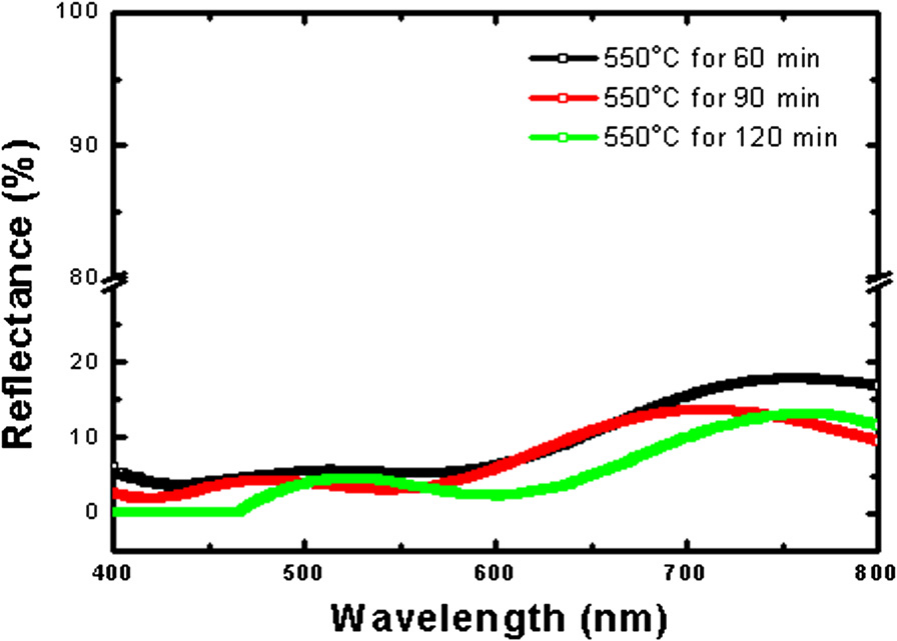
Reflectance spectra of ZnO nanowires grown for 60, 90, and 120 min, respectively.

In order to investigate the influence of the ZnO NWs on light scattering, the spectral dependence of the total reflectance of nanowire arrays was analyzed. Figure 4 displays the reflectance spectra of ZnO NWs with different growth times of 60, 90, and 120 min. We can observe that the silicon substrates covered by ZnO NWs have lower reflectance spectra in the range of 400 to 800 nm. This figure shows that the ZnO NWs with a growth time of 120 min have the lowest average reflectance of about 5.7% throughout the visible range (approximately 9.7% for 60 min and approximately 7.6% for 90 min). That is simply because it has been realized that ZnO NWs with strong alignment, high aspect ratio, and uniform distribution can effectively enhance the antireflection coatings (ARCs) by trapping light and leading to a broadband suppression in the reflection [17,18] Accordingly, we expect that longer ZnO NWs have a much higher chance for the incident photons interacting with the NWs' surfaces, and therefore, the absorption cross section would be considerably larger than the short ones as we increase the growth time.

**Figure 5:**
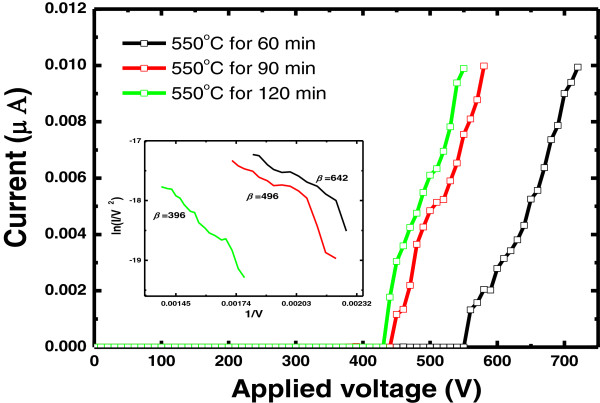
**Field emission characteristics of ZnO NWs.** They were grown for 60, 90, and 120 min, respectively. The inset shows Fowler-Nordheim plots of ln(*I*/*V*^2^) versus (1/*V*).

Figure 5 shows the field emission *I*-*V* plots for the ZnO nanowire with different growth times. Note that all samples show similar emission current–voltage (*I*-*V*) characteristics despite the different growth times. There are two different regions manifested in the *I*-*V* curve of all samples. In the low-voltage region, the emission current is low and seems to be independent of the applied voltage. Once the voltage is increased further, the emitted current increases dramatically and the turn-on voltages are 410, 440, and 550 V for growth times of 120, 90, and 60 min, respectively.

In order to analyze the emission behavior, the *I*-*V* characteristics of ZnO NWs are interpreted using the Fowler-Nordheim (FN) equation:

(1) $$J=\frac{aV^{2}}{\beta^{2}d^{2}\varphi }\exp\left(\frac{\mathrm{bd}\varphi^{3/2}}{\mathrm{\beta V}}\right)$$

where *J* is the current density, *V* is the applied voltage, *β* is the work function, *d* is the emitting distance, *β* is the field enhancement factor, and *a* and *b* are the constants. As shown in the inset of Figure 5, factor *β* in the FN equation represents the degree of field emission enhancement. For a nanostructured emitter, the *β* value is related to its work function, morphology, crystallinity, conductivity, and density. By assuming 5.2 eV as the work function value for ZnO NWs, field enhancement factors were calculated to be 642, 492, 396 for growth times of 60, 90, and 120 min, respectively [19-21]. The field emission properties of ZnO NWs are expected to improve as their length is increased, which can be qualitatively explained by the increase in the field enhancement factor *β* for long NWs. However, our experimental results contradict the anticipation. The phenomenon can be ascribed to the compensation by the increase of their diameter. Based on our experimental results, the growth time plays an important role in density and morphology control of ZnO NWs and thus modifies the optoelectronic properties for versatile devices.

## Conclusions

In summary, the vertical arrays of well-aligned *c*-axis orientation ZnO NWs have been synthesized on silicon substrate by VS growth mechanism at a relatively low growth temperature. By varying the growth time, we can adjust the areal density, length, and diameter of ZnO NWs and modify the structural and optoelectronic properties accordingly. PL spectra measured at room temperature exhibit a sharp UV peak and broad green band, corresponding to the NBE and defect-related emissions, respectively. When the growth time increased, the average diameter of NWs became larger and thus the surface-to-volume ratio became lower. Therefore, higher surface states of ZnO NWs with smaller diameters can be responsible for the origin of enhanced green emission. ZnO NWs with strong alignment and uniform distribution can also minimize the reflectance to 5.7% in the visible region. In addition, field emission features revealed that the growth time plays an important role in density- and morphology-controlled ZnO NWs. It is reasonable to expect that the ZnO NWs can be modified to meet the requirements for versatile optoelectronic devices.

## Abbreviations

NBE: Near-band-edge; PL: Photoluminescence; SEM: Scanning electron microscopy; XRD: X-ray diffraction.

## Competing interests

The authors declare that they have no competing interests.

## Authors’ contributions

H-IL designed and carried out the experiment and statistical analysis and participated in drafting the manuscript. S-YK supervised the research and revised the manuscript. Both authors read and approved the final manuscript.
